# Bias dependent variability of low-frequency noise in single-layer graphene FETs†

**DOI:** 10.1039/d0na00632g

**Published:** 2020-10-26

**Authors:** Nikolaos Mavredakis, Ramon Garcia Cortadella, Xavi Illa, Nathan Schaefer, Andrea Bonaccini Calia, Anton-Guimerà-Brunet, Jose A. Garrido, David Jiménez

**Affiliations:** Departament d'Enginyeria Electrònica, Escola d'Enginyeria, Universitat Autònoma de Barcelona Bellaterra 08193 Spain nikolaos.mavredakis@uab.es; Catalan Institute of Nanoscience and Nanotechnology (ICN2), CSIC, Barcelona Institute of Science and Technology Campus UAB, Bellaterra Barcelona Spain; Instituto de Microelectronica de Barcelona, IMB-CNM (CSIC) Esfera UAB, Bellatera Spain; Centro de Investigacion Biomedica en Red en Bioingenieria, Biomateriales y Nanomedicina (CIBER-BBN) Madrid Spain; ICREA Pg. Lluis Companys 23 08010 Barcelona Spain

## Abstract

Low-frequency noise (LFN) variability in graphene transistors (GFETs) is for the first time researched in this work under both experimental and theoretical aspects. LFN from an adequate statistical sample of long-channel solution-gated single-layer GFETs is measured in a wide range of operating conditions while a physics-based analytical model is derived that accounts for the bias dependence of LFN variance with remarkable performance. LFN deviations in GFETs stem from the variations of the parameters of the physical mechanisms that generate LFN, which are the number of traps (*N*_tr_) for the carrier number fluctuation effect (Δ*N*) due to trapping/detrapping process and the Hooge parameter (*α*_H_) for the mobility fluctuations effect (Δ*μ*). Δ*N* accounts for an M-shape of normalized LFN variance *versus* gate bias with a minimum at the charge neutrality point (CNP) as it was the case for normalized LFN mean value while Δ*μ* contributes only near the CNP for both variance and mean value. Trap statistical nature of the devices under test is experimentally shown to differ from classical Poisson distribution noticed at silicon-oxide devices, and this might be caused both by the electrolyte interface in GFETs under study and by the premature stage of the GFET technology development which could permit external factors to influence the performance. This not fully advanced GFET process growth might also cause pivotal inconsistencies affecting the scaling laws in GFETs of the same process.

## Abstract 2

Low-frequency noise variability is for the first time examined in single-layer graphene transistors while an analytical compact model demonstrating an outstanding performance is proposed.

## Graphical abstract

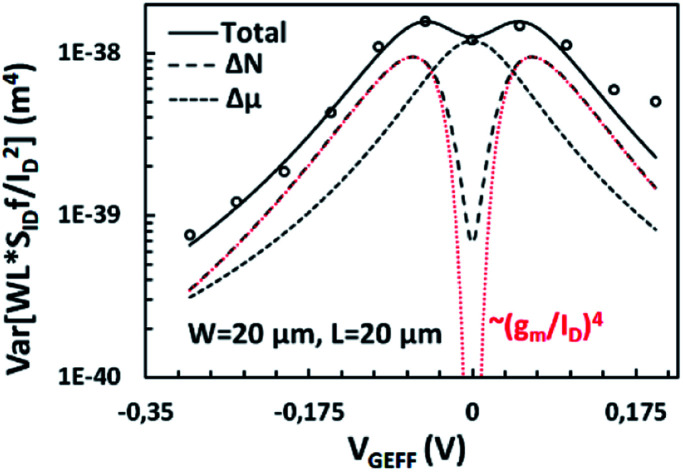

## Introduction

Nowadays, the limitations on advanced CMOS technologies and the predictions for deceleration of Moore's law, have led both the scientific community and semiconductor industry to turn their attention at the development of emerging technologies based on 2-Dimensional (2D) materials such as graphene.^1,2^ Graphene's extraordinary characteristics such as carrier mobilities up to 2 × 10^5^ cm^2^ (V s)^−1^ and saturation velocities of 4 × 10^7^ cm s^−1^ have placed graphene transistors (GFET) as a quintessential prospective for future applications.^3^ Despite the fact that the lack of bandgap in graphene due to its semimetal nature makes single-layer (SL) GFETs unsuitable for digital electronics, there has been an enormous increase of analog and RF circuits designed with GFET technology such as: frequency multipliers,^4,5^ voltage control^6,7^ and ring oscillators^8^ as well as terahertz detectors.^9–11^ Moreover, GFETs are also widely used and tremendously improve the performance of chemical–biological sensors and optoelectronic devices.^12–17^

It is not enough for recently developed GFET technologies just to exhibit optimal performance, but they should also be reliable and consistent with CMOS ones since the majority of industry aims to develop new GFET processes based on their pre-existing infrastructures designed for silicon devices.^18,19^ Variability issues are of outmost importance in advanced semiconductor technologies. Regarding GFETs, the thorough study of such effects is crucial for the transition from immature technologies and prototyped devices mainly fabricated in research labs to large-scale wafer production which will lead to a boost of graphene-based applications and products. There are two sources of variabilities in graphene: (a) environmental effect variabilities such as interface traps and (b) material imperfection variabilities such as edge disorders.^18^ In this work we focus on low-frequency noise (LFN) variability for SL GFETs mainly derived from interface trap statistics which, according to our knowledge, is for the first time investigated. LFN's contribution to the aforementioned circuits is very crucial as it can be upconverted to deleterious phase noise in such high frequency applications.^4–11^ In addition, it can affect the sensitivity of sensors^12–17^ while LFN deviations can also be proved useful for such sensing applications.^18^

Moreover, LFN examination can provide crucial information as far as the quality of the devices and their interfaces is concerned.^20,21^

Three main effects are considered responsible for the generation of LFN in semiconductor devices and consequently GFETs; firstly, the carrier number fluctuation mechanism (Δ*N*), secondly the mobility fluctuation mechanism (Δ*μ*) and finally the series resistance (*R*_c_) contribution (Δ*R*). Δ*N* model stems from the trapping/detrapping process at semiconductor devices.^22,23^ In more detail, a free carrier can be captured by an active trap near the dielectric interface and within a few *kT* from the Fermi level and then emitted back at the conduction path, and as a result a Random Telegraph Signal (RTS) is generated which corresponds to a Lorentzian Power Spectral Density (PSD). For transistors with channels larger than a few hundred nanometers, the number of active traps is high and consequently the superposition of the generated Lorentzian spectra can result in LFN PSDs inversely proportional to frequency under the condition that the traps are uniformly distributed. This is also known as 1/*f* noise and was first proposed by McWhorter.^24^ Minimization of device dimensions in advanced CMOS technology nodes has led both the LFN mean value and variance to be dominated by RTS^25–27^ but this is not yet the case in GFETs. Δ*μ* model occurs due to fluctuations of the bulk mobility and is described by the empirical Hooge expression^28^ while Δ*R* one is caused by *R*_c_ contribution especially at high current regimes. Several physics-based models, simpler or more analytical ones, are available in literature describing both LFN mean value^29–33^ and variance^34–40^ in CMOS transistors. Most of the LFN variance models focus on its area dependence for short channel CMOS devices where RTS prevail^34–37^ while bias dependence is also analyzed in some of them.^38–40^ Characterization and modeling of the standard deviation of the natural logarithm of LFN is also very common in bibliography since LFN deviations follow a log-normal distribution.^25,27,35–37,39,40^

A lot of research has also been conducted regarding LFN in GFETs^41–55^ and the findings agree that the same mechanisms (Δ*N*, Δ*μ*, Δ*R*) are responsible for the generation of LFN. In fact, it has been shown that the nature of LFN in GFETs strongly relies upon the number of layers of the device.^47^ In transistors with many layers, volume noise (Δ*μ*) prevails while the fewer the layers the more significant the surface LFN (Δ*N*) becomes. In this work, SL GFETs are governed by trapping/detrapping mechanism which causes an M-shape gate-bias dependence of output LFN divided by squared drain current (*S*_*I*_D__*f*/*I*_D_^2^), referred to 1 Hz, with a minimum at the charge neutrality point (CNP). Residual charge, which dominates at the CNP, can proportionally increase the LFN minimum. Similarly, non-homogeneous charge density at the channel, caused by a relative high drain voltage, can also increase the LFN minimum.^53^ Δ*μ* model can also contribute to LFN near the CNP always with a Λ-shape trend even at SL GFETs while Δ*R* has been observed at higher current regime where *R*_c_ is important.^53–55^ LFN in GFETs can be reduced after electron-beam irradiation^49^ while the same can be achieved with the usage of substrates such as boron nitride.^50,51^ A number of models have been proposed to describe the behavior of LFN mean value on GFETs but most of them^43,45,46^ are based on a simple approximation for Δ*N* model taken from CMOS devices^26,29,31^ which introduces an ∼(*g*_m_/*I*_D_)^2^ trend of *S*_*I*_D__*f*/*I*_D_^2^ LFN. The latter approach can only be functional under uniform channel conditions.^54,55^ Recently, a complete physics-based LFN model was proposed^53–55^ which is valid in all operating regions since it accounts for all non-homogeneities of the device.

While there is a significant number of works regarding LFN mean value modeling in GFETs, no studies are available that deal with LFN variability in these devices even though it is equally significant to its mean value. The large deviations of LFN observed in GFETs, makes it urgent to develop statistical LFN models to investigate the physics behind this variability. In the present work, such an analysis that combines both the experimental characterization of LFN variance data and the derivation of a new physics-based statistical compact model, is proposed for the first time. The proposed model reveals the relation of LFN variability with the deviation of the parameters of the fundamental physical generators of LFN in GFETs (Δ*N*, Δ*μ*). As it is known from CMOS technology, LFN variance is connected with operating conditions in larger devices,^39,40^ which is the case for the GFETs under study in the present analysis. The proposed model is based on the recently established chemical-potential based one regarding LFN mean value^53^ as well as *CV*–*IV* behavior.^56–58^ For more details on the *CV*–*IV*–LFN model see ESI A (Fig. S1).† The principal idea for the LFN mean value model calculation was to divide the device channel into microscopic uncorrelated local noise sources. The local noise PSD that originates from each LFN mechanism was then calculated and integrated from source to drain in order to analytically evaluate the contributions from Δ*N*, Δ*μ* and Δ*R* LFN; by adding these contributions the total LFN PSD is obtained.^33^ Similarly, in the present work, the variance is calculated locally for each LFN mechanism by applying fundamental laws of statistics.^39,40^ Then, the integration along the channel leads to an analytical compact solution with the help of the chemical-potential based model mentioned above. The derived model describes very accurately the experimental data and as in CMOS devices,^39,40^ Δ*N* and Δ*μ* variance present a bias dependence similar to their means. Thus, Δ*N* variance is responsible for an M-shape of total LFN variance centered at the CNP, while Δ*μ* variance is more significant near the CNP. The proposed LFN variance compact model can be easily implemented in Verilog-A and annexed at the chemical-potential based model mentioned above ^53–58^ and then included in circuit simulators.

Trapping/detrapping mechanism that generate LFN is influenced by some additional factors at the solution-gated (SG)-SL GFETs under study^59^ resulting in a trap distribution different than Poisson which is the case in silicon-oxide devices.^27,35–38^ More particularly, trapping/detrapping can occur either near the surface of the polyimide substrate or at the graphene–electrolyte interface where processes of association/dissociation with charged moieties can take place.^60–63^ In addition, GFET technologies are still in a non-optimal stage and thus, extrinsic traps might be generated during the fabrication process affecting the trap statistics behavior.

## Results and discussion

**Fig. 1:**
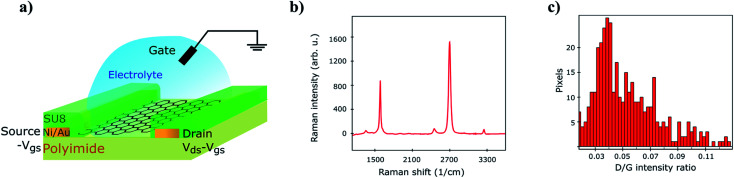
(a) Schematic of a SG-SL GFET. (b) Average Raman spectrum over the whole graphene channel and (c) histogram of D/G ratio representative of the defects density distribution.

On wafer LFN and *IV* measurements were conducted at top SG-SL GFETs^59^ at three different device geometries; (a) *W* = 100 μm/*L* = 100 μm, (b) *W* = 50 μm/*L* = 50 μm and (c) *W* = 20 μm/*L* = 20 μm where *W*, *L* are the width and length of the device respectively. Top gate voltage was swept from strong p-type to strong n-type region including the CNP with a step of 5 mV while drain voltage was constant, *V*_DS_ = 50 mV. Arrays of SG-SL GFETs were fabricated (see Experimental data section for more details on fabrication and measurements procedures) and thus, a significant number of samples were measured for each available geometry in order to have adequate data to characterize LFN statistics. In more detail, the analysis took place at around 48–50 samples from geometry (a), 25–28 samples from geometry (b) and 23–25 samples from geometry (c) after the exclusion of outliers. The reason why the number of samples is not constant for each geometry is that some measurements might behave as outliers in some specific operating conditions but not in the whole range. While *I*_D_ and *S*_*I*_D__ were measured for 100 top gate voltage values, only 11 of them were chosen for the LFN variability analysis in order to speed up the process. These values were extended from high-to low-current regime both at p- and n-type regions in order to permit the thorough study of the LFN variance at all the operating conditions. The schematic of the device under test is shown in Fig. 1a where the graphene channel, the metal contacts, the SU8 passivation, the electrolyte gate and the reference electrode are shown. Fig. 1b illustrates the average Raman spectrum from 400 points in the *W* = 20 μm/*L* = 20 μm GFET area after the transfer of graphene (see Experimental data section). Fig. 1c shows a histogram representing the D/G ratio for each of the measured spectra, indicating the rather low density of defects in the graphene lattice.

### *IV* model validation

**Fig. 2:**
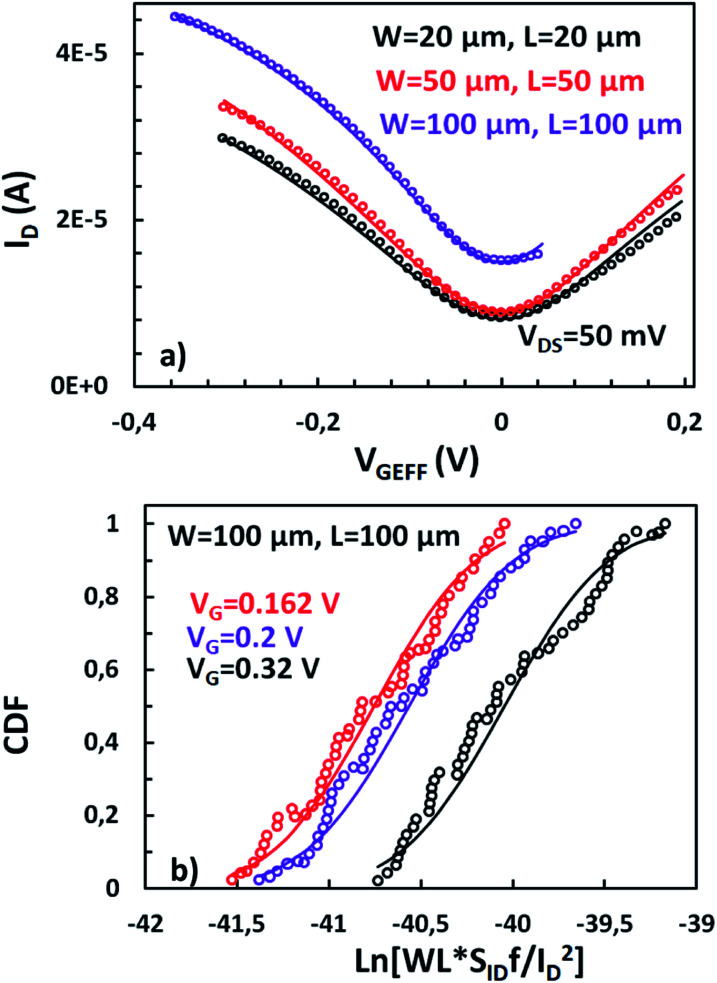
(a) Drain current *I*_D_*vs.* top gate voltage overdrive *V*_GEFF_, for GFETs with *W*/*L* = 20 μm/20 μm, 50 μm/50 μm and 100 μm/100 μm. Markers: measured data, solid lines: model. (b) Cumulative distribution function (CDF) of natural logarithm of normalized LFN ln(*WLS*_*I*_D__*f*/*I*_D_^2^), referred to 1 Hz, for GFET with *W*/*L* = 100 μm/100 μm, shows a log-normal distribution. Markers: extracted CDF, solid lines: theoretical CDF of normal distribution of ln(*WLS*_*I*_D__*f*/*I*_D_^2^).

The first step towards the modeling of LFN mean and variance is the extraction of physical parameters related to their stationary response. In Fig. 2a, *IV* model is validated^56–58^ for the three GFET geometries under test. The transfer characteristics (drain-source current *I*_D_*vs.* effective gate voltage *V*_GEFF_) are shown for all regimes of operation, near and away the CNP, with *V*_GEFF_ calculated as the gate voltage (*V*_G_) minus the voltage at the CNP (*V*_CNP_). Fig. 2a also presents the fitting from the chemical-potential based model,^56–58^ showing a close match with the experimental data. The fundamental parameters of the *IV* model such as mobility *μ*, top gate capacitance *C*_top_, flat band voltage *V*_GSO_, residual charge *ρ*_0_ and contact resistance *R*_c_ are extracted and presented in Table 1. Derived parameters from every GFET are very close, apart from *μ* and *V*_GSO_ which are quite heightened for the larger device, which is indicative of the elevated *I*_D_ data observed there. Also, *R*_c_ for the medium sized device is a little decreased.

**Table 1:** *IV*–LFN (mean value–variance) model parameters

Parameter	Units	*W*/*L* = 20 μm/20 μm	*W*/*L* = 50 μm/50 μm	*W*/*L* = 100 μm/100 μm
*μ*	cm^2^ (V s)^−1^	3500	3800	7000
*C*_top_	μF cm^−2^	2	2	2
*V*_GSO_	V	0.278	0277	0.33
*ρ*_0_	cm^−2^	3.29 × 10^11^	3.29 × 10^11^	3.43 × 10^11^
*R*_c_	Ω	400	320	380
*N*_T_	eV^−1^ cm^−3^	2.2 × 10^19^	4.93 × 10^19^	1.74 × 10^20^
*α*_H_	—	5.6 × 10^−4^	1.5 × 10^−3^	1.12 × 10^−2^
*S*_Δ*R*_^2^	Ω^2^ Hz^−1^	3 × 10^−4^	4 × 10^−5^	1.8 × 10^−5^
*N*_tcoeff_	—	2.7 × 10^3^	2.5 × 10^5^	3.4 × 10^6^
*N*_*α*_H__	—	1.19 × 10^13^	3.5 × 10^11^	3.6 × 10^10^

### LFN mean value model

**Fig. 3:**
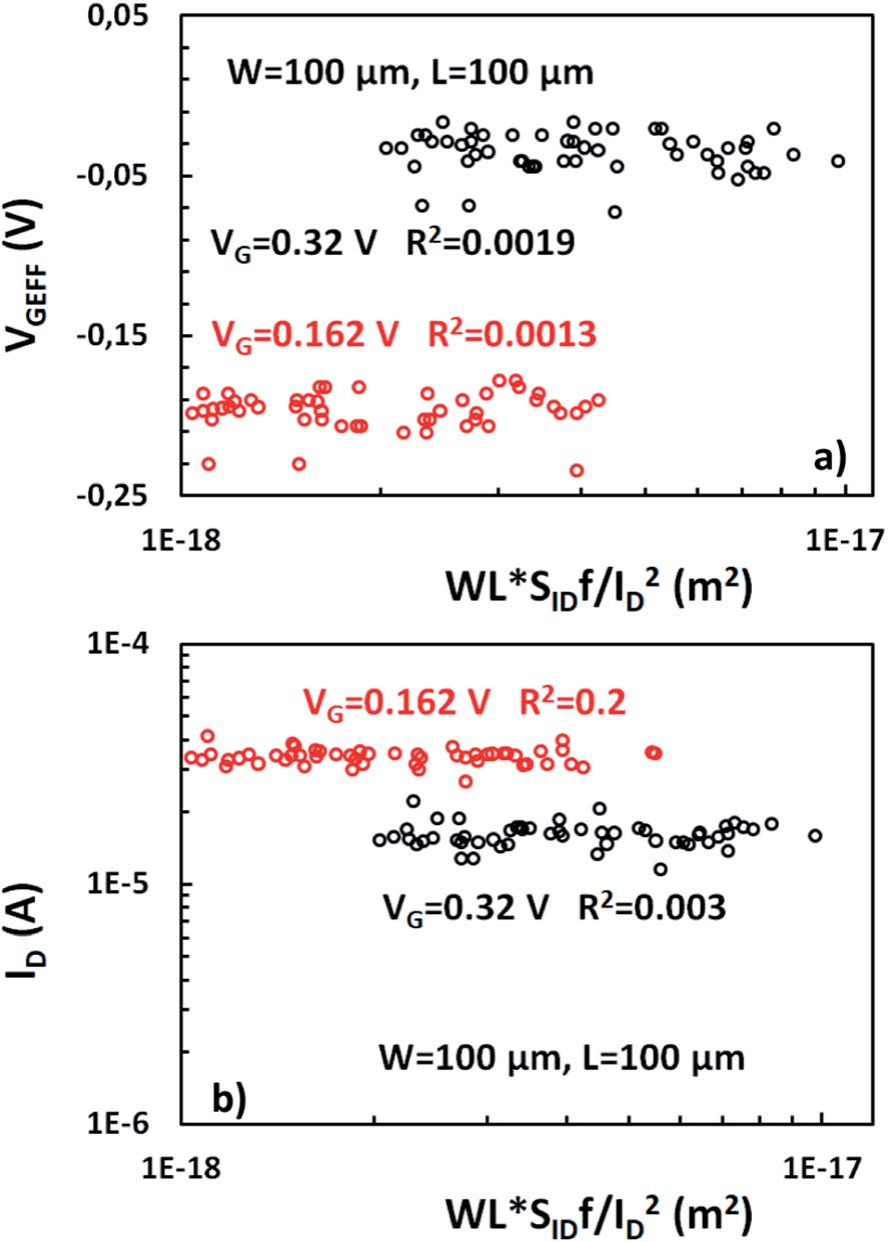
(a) Variability of *WLS*_*I*_D__*f*/*I*_D_^2^, referred to 1 Hz, is much higher and uncorrelated with variability of (a) *V*_GEFF_ and (b) *I*_D_ for GFET with *W*/*L* = 100 μm/100 μm.

**Fig. 4:**
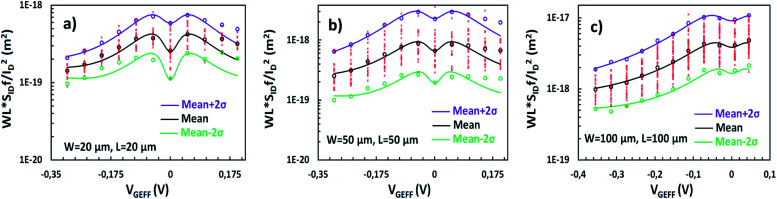
Normalized LFN *WLS*_*I*_D__*f*/*I*_D_^2^, referred to 1 Hz, *vs.* top gate voltage overdrive *V*_GEFF_, for GFETs with (a) *W*/*L* = 20 μm/20 μm, (b) 50 μm/50 μm and (c) 100 μm/100 μm. Measured noise from all available samples: star markers, measured ln-mean noise and its ±2-sigma deviation: open circle markers, mean and ±2-sigma deviation model: lines (mean data and model: black, +2-sigma deviation data and model: purple, −2-sigma deviation data and model: green).

As described before, Δ*N*, Δ*μ*, Δ*R* are the main generators of LFN in GFETs. Regarding LFN variance, the first two are going to be investigated in this work. LFN normalized by the area over squared drain current (*WLS*_*I*_D__*f*/*I*_D_^2^), at 1 Hz, is widely used in literature for the study of LFN variance. The reason for using this normalization is that the variance of this term presents a ∼1/(*WL*) dependence.^35,36,39,40^ In Fig. 2b, this form of depiction of LFN data is shown to follow a log-normal distribution (*σ*[ln(LFN)] = √ln(1 + Var(LFN)/*E*^2^(LFN)), where *σ* is the standard deviation and *E* the mean value^37,40^) for the first time in GFETs. Cumulative distribution function (CDF) of the natural logarithm of *WLS*_*I*_D__*f*/*I*_D_^2^ for the 100 μm/100 μm GFET is presented at three different *V*_GEFF_; markers present the extracted CDF of the random variable *X* = ln[*WLS*_*I*_D__*f*/*I*_D_^2^] while the solid lines the theoretical CDF under the consideration that *X* is normally distributed. The precise fitting among markers and lines confirms that *WLS*_*I*_D__*f*/*I*_D_^2^ data indeed follow a log-normal distribution. Fig. 3a and b indicate the correlation of the *WLS*_*I*_D__*f*/*I*_D_^2^ variability with *V*_GEFF_ (or *V*_CNP_ equivalently) and *I*_D_ variabilities, respectively for the same device. It is evident that this correlation is very weak, and this observation proves that LFN variance is not related to the variability of the *IV* characteristics but is mostly due to trap statistics as well as Hooge parameter variations as it will be shown later. See ESI B (Fig. S2)† for the equivalent plots as Fig. 2b and 3a for the rest of the GFET areas. Therefore, for the derivation of the LFN variance model, it is crucial to first determine the parameters of the LFN mean value model that are sensitive to variations. These are the number of traps *N*_tr_ from Δ*N* effect and Hooge parameter *α*_H_ from Δ*μ* one. Fluctuations of the two aforementioned parameters from sample to sample lead to different LFN magnitudes and consequently cause LFN variance. Thus, the only way to explicitly model this variance is to examine how the deviation of these parameters affect the statistical behavior of the two mechanisms that generate LFN. The *WLS*_*I*_D__*f*/*I*_D_^2^ PSD locally in the device's channel for a slice Δ*x*, for both mechanisms, is given by:^53^1
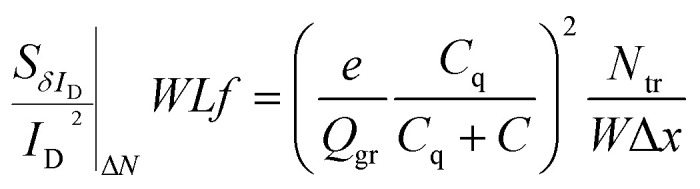
2
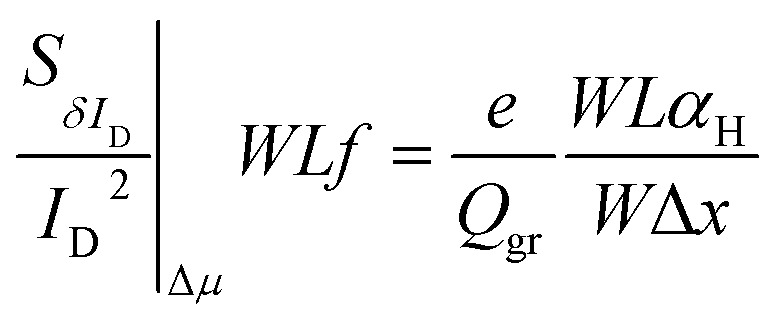
respectively, where *N*_tr_ = *WLN*_t_ = *KTλN*_T_^31,32^ is the number of active traps, *N*_t_ is the trap density in (cm^−2^) and *N*_T_ is the volumetric trap density in (eV^−1^ cm^−3^) which is used as a LFN mean value model parameter.^53^*K* is the Boltzman constant, *T* is the absolute temperature, *λ* ≈ 0.1 nm is the tunneling attenuation distance, *e* is the electron charge, *Q*_gr_ is the graphene charge stored in the quantum capacitance *C*_q_, *C* = *C*_top_ + *C*_back_ is the sum of top and back interface capacitances, *α*_H_ is the unitless Hooge parameter which is also used as a LFN mean value model parameter^53^ and *f* is the frequency. See ESI A (Fig. S1)† for more details on the definition of different quantities. To obtain the total LFN PSDs, integration of the local PSDs derived above with an integral variable change from length *x* to chemical potential *V*_c_ should take place.^53–55^ The SG-SL GFETs which are examined in this study are long-channel devices operating at low electric field region due to small drain voltage and thus, Velocity Saturation (VS) effect^54^ is not expected to contribute to LFN variance. Consequently, VS effect is ignored in the derivation of the model that follows in order to keep the expressions simple. The complete model including VS contribution to LFN variance is presented in ESI C (see eqn (S6)–(S12), Fig. S3).† It is confirmed that VS is negligible under low electric field conditions while it can increase variance at high electric fields. LFN mean value model is validated with experimental data, averaged in a bandwidth 1–30 Hz, in Fig. 4 for all the GFETs under test. *WLS*_*I*_D__*f*/*I*_D_^2^ ln-mean data with black circular markers and LFN mean value model^53^ with solid black lines are shown for the *W*/*L* = 20 μm/20 μm GFET in Fig. 4a, for the *W*/*L* = 50 μm/50 μm GFET in Fig. 4b and for the *W*/*L* = 100 μm/100 μm GFET in Fig. 4c*vs. V*_GEFF_ with very consistent results for all the devices and for all regions of operation. The logarithmic (ln)-mean values are used for better accuracy due to log-normal distribution of LFN. The LFN data for the total of the measured samples are also shown with smaller red markers. Δ*μ* model is significant near the CNP and from there the *α*_H_ parameter can be extracted, Δ*N* LFN is responsible for the M-shape of *WLS*_*I*_D__*f*/*I*_D_^2^ and thus, *N*_T_ parameter can be extracted from fitting the Δ*N* model of LFN generation. Finally, the contribution from Δ*R* can be identified at higher gate voltage values, where *S*_Δ*R*_^2^ can be extracted.^55^ The LFN mean value model parameters are presented in Table 1. *N*_T_ and *α*_H_ increase with the area of the GFETs, with the greatest increment observed in the largest devices (*W*/*L* = 100 μm/100 μm), where they double with respect to the medium sized devices (*W*/*L* = 50 μm/50 μm). Oppositely, *S*_Δ*R*_^2^ reduces with the GFET channel area. The reason for this deviation of LFN mean value parameters is not well understood but GFET technologies are not as mature as CMOS for example, and this might cause such deviations even for parameters of the same technology. The ensuing LFN statistical analysis will confirm that Δ*N* and Δ*μ* models define LFN variance similarly as its mean value.

### LFN variance model

Variations in the number of active traps (*N*_tr_) can definitely induce variations in the Δ*N* contribution to LFN. Similarly, variations in the *α*_H_ parameter can produce variability in the effect of Δ*μ* on LFN. In this section, an analytical LFN variance model for each of the two LFN generation mechanisms will be derived. First, LFN variance will be calculated locally in the channel and since the local noise sources are considered uncorrelated,^33,53^ integration from source to drain can provide the total LFN variance by adding all the local contributions. Estimating variance locally ensures that each Δ*I*_D_/*I*_D_ deviation caused by a fluctuation (such as a specific trap) at any infinitesimal area will contribute independently.^25,37,39,40^ Local variance represents the relative variation induced by each local noise source. Oppositely, only under uniform channel conditions where local variance would be equal throughout the whole channel, it could be taken out of the integral. But this is not the case in this work since all non-homogeneities are taken into account in order to implement a complete model. Fundamental statistics theory gives:3
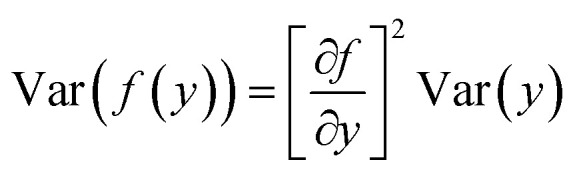
where in this study *y* = *N*_tr_, *α*_H_ for Δ*N*, Δ*μ* models respectively and *f*(*N*_tr_, *α*_H_) = *Λ*(*x*) (*N*_tr_, *α*_H_).

### Δ*N* LFN variance

The total *WLS*_*I*_D__*f*/*I*_D_^2^ Δ*N* is calculated by integrating eqn (1) along the channel:^33,53^4
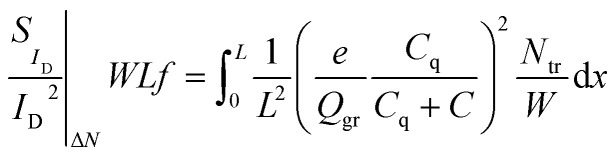


To calculate the variance of eqn (4):5
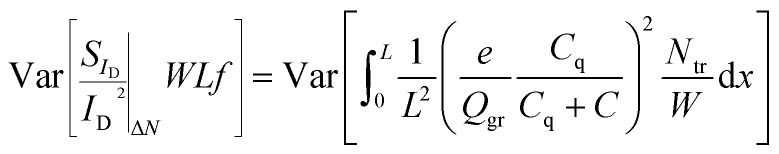


It can be easily proved that since the local noise sources are uncorrelated and thus independent, the following expression is valid:6
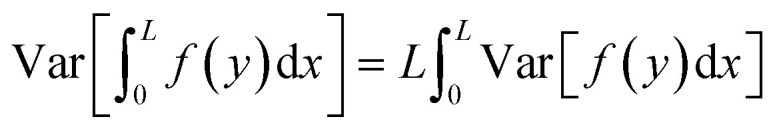


See ESI D† for the mathematical proof of eqn (6). Because of eqn (3) and (6), variance of eqn (5) can enter the integral as:7
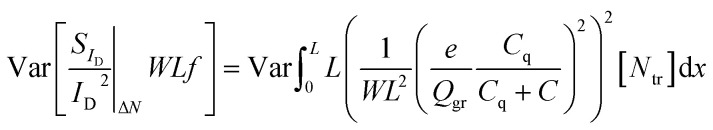
where the quantity inside the integral corresponds to the local variance. In silicon-oxide devices number of traps are known to follow Poisson distribution^27,35–38^ which means that its variance equals to its mean value, Var[*N*_tr_] = *N*_tr_ = *WLKTλN*_T_. It will be shown later that this is not the case in SG-SL GFETs of this work but it rather is Var[*N*_tr_] = *N*_tcoeff_*WLKTλN*_T_, where *N*_tcoeff_ is used as a fitting LFN variance model parameter. According to the latter and if the integral variable is changed from *x* to *V*_c_:^53–58^8
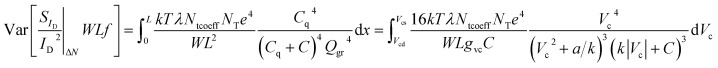
and if eqn (8) is solved analytically, eqn (9) is derived.9
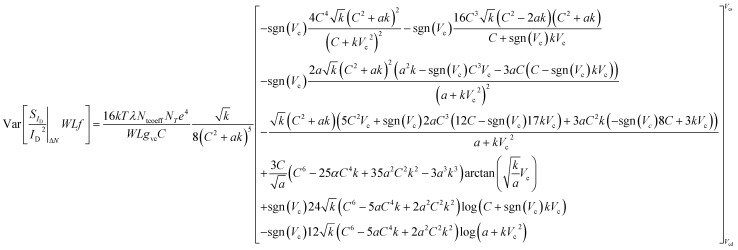
*g*_vc_ is a normalized drain current term,^53–58^*α* = 2*ρ*_0_*e* is a residual charge related expression^55–58^ and *k* is a coefficient.^53–58^ For more details on definitions see ESI A.†eqn (9) predicts an inversely proportional relation of the *WLS*_*I*_D__*f*/*I*_D_^2^ Δ*N* variance model to the area of the device ∼1/(*WL*).

### Δ*μ* LFN variance

Following a procedure similar as in Δ*N* case, the total *WLS*_*I*_D__*f*/*I*_D_^2^ Δ*μ* variance is calculated by integrating eqn (2) along the channel:^33,53^10
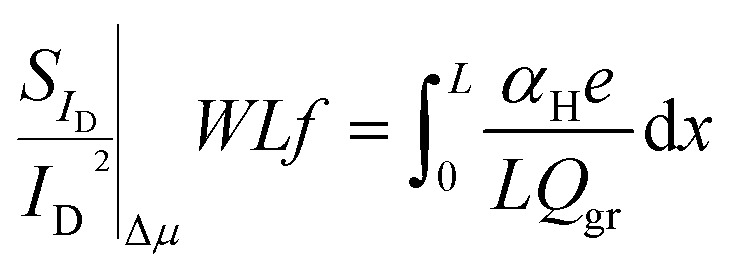


Variance of eqn (10) is calculated after taking into consideration eqn (3) and (6) as in Δ*N* case since local noise sources are uncorrelated:11
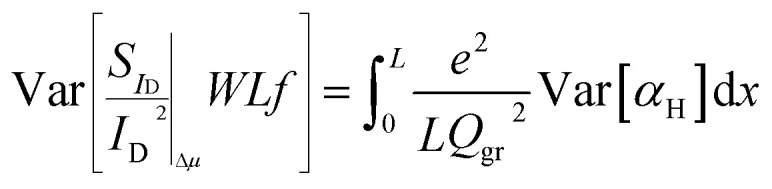


No information is available on literature regarding Var[*α*_H_] and thus, in order to achieve a similar scaling with Δ*N WLS*_*I*_D__*f*/*I*_D_ LFN variance model ∼1/(*WL*), the following is assumed: Var[*α*_H_] = *α*_H_(*N*_*α*_H__*WL*)^−1^ where *N*_*α*_H__ is a specific density used as a fitting LFN variance model parameter. According to the latter and if the integral variable is changed from *x* to *V*_c_:^53–58^12
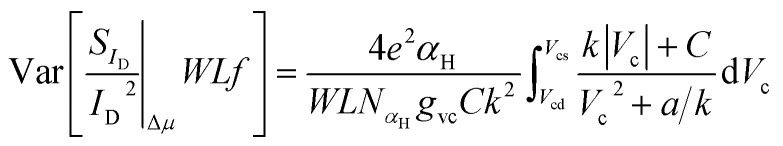
and if eqn (12) is solved analytically:13
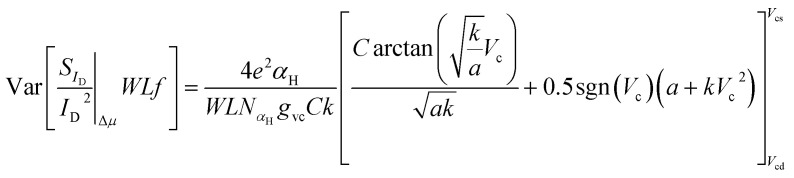


### Total LFN variance

The total *WLS*_*I*_D__*f*/*I*_D_^2^ variance can be calculated as:14
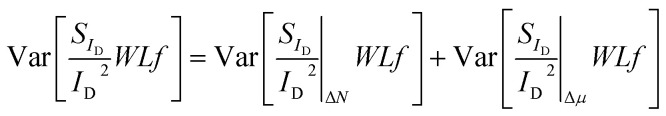
under the approximation that Δ*N* and Δ*μ* models are uncorrelated. Even though this is not completely accurate, there are distinguished regions where each of these effects is dominant (Δ*μ* at the CNP and Δ*N* at the peaks of M-shape dependence) and thus the aforementioned independency can be assumed without significant error. Eqn (9), (13) and (14) formulate the new compact statistical LFN model. As shown before, *WLS*_*I*_D__*f*/*I*_D_^2^ follows a log-normal distribution as it can be observed by the CDFs illustrated in Fig. 2b and in ESI B (Fig. S2a and b).† the fundamental expression for this distribution is:^37,39^15
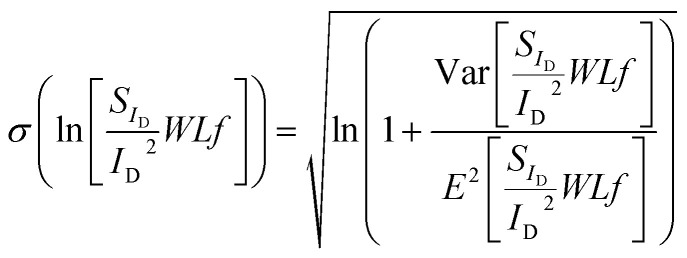
where *σ*(ln[*WLS*_*I*_D__*f*/*I*_D_^2^]) is the standard deviation of the natural logarithm of *WLS*_*I*_D__*f*/*I*_D_^2^ widely used in bibliography,^25,27,35–37,39,40^*E* denotes the LFN mean value model and the ratio of variance with squared mean *WLS*_*I*_D__*f*/*I*_D_^2^ is defined as normalized variance.^25,27,35–37,39,40^ The model in eqn (15) follows the scaling dependence of ref. 37, which is ∼√ln[1 + *K*/(*WL*)] and for larger devices turns to ∼√1/(*WL*) where *K*/(*WL*) is the normalized variance established before.^37^

### Experimental validation of the LFN variance model

The qualitative performance of the new derived LFN variance model is verified with the data from SG-SL GFETs under test, as it will be illustrated in the rest of this section. Initially, as it was mentioned before, ln-mean of *WLS*_*I*_D__*f*/*I*_D_^2^ data is calculated for every device under test from all the available samples and used in the verification of the LFN mean value model as in Fig. 4. Afterwards, standard deviation *σ*(ln[*WLS*_*I*_D__*f*/*I*_D_^2^]) data can be easily extracted from the natural logarithms of all the *WLS*_*I*_D__*f*/*I*_D_^2^ samples again for each available GFET. This process is followed for the derivation of *σ*(ln[*WLS*_*I*_D__*f*/*I*_D_^2^]) instead of eqn (15) since noise measurements are very sensitive and thus the calculation of normalized variance ratio Var[*WLS*_*I*_D__*f*/*I*_D_^2^]/*E*^2^[*WLS*_*I*_D__*f*/*I*_D_^2^] contained in eqn (15) is not very consistent because of the small numbers both in numerator and denominator. Then, *WLS*_*I*_D__*f*/*I*_D_^2^ variance can be estimated through eqn (15) since *σ*(ln[*WLS*_*I*_D__*f*/*I*_D_^2^]) is already known, by using the ln-mean in the denominator of normalized variance. In Fig. 4, ±2σ standard deviation of *WLS*_*I*_D__*f*/*I*_D_^2^ (*σ* = √Var) is also shown both for the model and experimental data with purple (+2*σ*) and green (−2*σ*) solid lines and markers respectively. The model captures accurately the dispersion of the data and its bias dependence, confirming the consistency between LFN mean value and variance models for all the three GFETs examined.

**Fig. 5:**
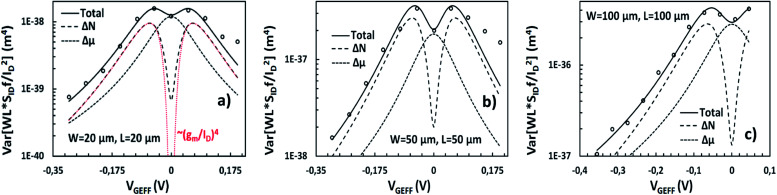
Variance of normalized LFN Var[*WLS*_*I*_D__*f*/*I*_D_^2^], referred to 1 Hz, *vs.* top gate voltage overdrive *V*_GEFF_, for GFETs with (a) *W*/*L* = 20 μm/20 μm, (b) 50 μm/50 μm and (c) 100 μm/100 μm. Markers: measured data, solid lines: total model, dashed lines: individual contributions (Δ*N*, Δ*μ*). Simplified LFN variance model ∼(*g*_m_/*I*_D_)^4^ which considers a homogeneous channel is shown with red dashed lines for *W*/*L* = 20 μm/20 μm GFET (a) for comparison reasons.

**Fig. 6:**
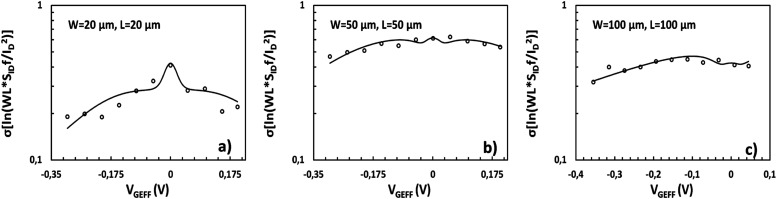
Standard deviation of natural logarithm of normalized LFN *σ*[ln(*WLS*_*I*_D__*f*/*I*_D_^2^)], referred to 1 Hz, *vs.* top gate voltage overdrive *V*_GEFF_, for GFETs with (a) *W*/*L* = 20 μm/20 μm, (b) 50 μm/50 μm and (c) 100 μm/100 μm. Markers: measured data, solid lines: total model.

*WLS*_*I*_D__ *f*/*I*_D_^2^ variance and *σ*(ln[*WLS*_*I*_D__*f*/*I*_D_^2^]) are depicted in Fig. 5 and 6 respectively for the *W*/*L* = 20 μm/20 μm GFET in Fig. 5a and 6a, for the *W*/*L* = 50 μm/50 μm GFET in Fig. 5b and 6b and for the *W*/*L* = 100 μm/100 μm GFET in Fig. 5c and 6c*vs. V*_GEFF_. Experimental data are represented with markers while the total model with solid lines. Dashed and dotted lines in Fig. 5 stand for the Δ*N* and Δ*μ* variance contributions, respectively. This representation confirms that the model precisely captures the experimental data for both *WLS*_*I*_D__*f*/*I*_D_^2^ variance and *σ*(ln[*WLS*_*I*_D__*f*/*I*_D_^2^]) in the whole range of operation for every GFET under investigation.

The Δ*N* and Δ*μ* models shown in Fig. 5 prove that these effects act similarly as in the LFN mean value model, Δ*N* effect is responsible for the M-shape of *WLS*_*I*_D__*f*/*I*_D_^2^ variance as it was for the *WLS*_*I*_D__*f*/*I*_D_^2^ mean value while Δ*μ* contributes near the CNP as it did for the LFN mean value model while it retains its Λ shape trend. The new LFN variance model shows a deviation from the experimental data at high n-type conduction regime probably due to Δ*R* contribution which is not included in this study. For comparison reasons, a LFN variance model based on ∼(*g*_m_/*I*_D_)^2^ approximation which considers a uniform channel,^26,29,31^ is shown in Fig. 5a with red dashed line. This approach estimates an ∼(*g*_m_/*I*_D_)^4^ dependence of LFN variance. For the analysis of the derivation of this expression see ESI E.† As it was expected, it gives acceptable results away from the CNP but it is incapable of capturing LFN variance near the CNP where the non-homogeneity of the device is more intense even for small *V*_DS_ values.^53^ These non-homogeneities can be easily detected from the illustration of the Δ*N* LFN contribution to *WLS*_*I*_D__*f*/*I*_D_^2^ variance throughout the channel where, for *V*_GEFF_ values away from the CNP, local variance is constant along the channel. Oppositely, near the CNP a steep dip is noticed in the middle of the channel in which the exact CNP is located under low *V*_DS_. For the above observations see ESI F (Fig. S4).† The experimental data were measured at *V*_DS_ = 50 mV, but it is quite certain that the inconsistency of the ∼(*g*_m_/*I*_D_)^4^ term would be more significant for higher *V*_DS_ values.

*N*_tcoeff_, *N*_*α*_H__ parameters of Δ*N* and Δ*μ* effects respectively, are extracted and shown in Table 1. *N*_*α*_H__ is calculated from the CNP while *N*_tcoeff_ is then adjusted to fit the M-shape. It is clear that *N*_tcoeff_ values for all GFETs are far from unity which means that *N*_tr_ does not follow a Poisson distribution as in silicon-oxide devices. As stated before, this might be both due to the nature of the traps generated in graphene–electrolyte interface^43,60–63^ and because of process-induced extrinsic traps of the specific SG-SL GFETs. In order to prove the validity of the obtained *N*_tcoeff_, *N*_*α*_H__ values, a thorough analysis was conducted where the LFN mean value model parameters (*N*_T_–*N*_tr_, *α*_H_) were extracted for each of the measured samples for all available GFET areas. The variance of these parameters as well as their ln-mean value were then estimated, allowing to derive the LFN variance model parameters *N*_tcoeff_, *N*_*α*_H__, given that Var[*N*_tr_] = *N*_tcoeff_*WLKTλN*_T_ and Var[*α*_H_] = *α*_H_(*N*_*α*_H__*WL*)^−1^. These values are proven to be identical with the ones extracted from Fig. 5 and shown in Table 1, which provide the best possible fitting of the model. This result confirms that charge traps do not present a Poisson distribution in the GFETs under study. For the complete analysis see ESI G (Fig. S5 and Table S1).† As it was mentioned before, *WLS*_*I*_D__*f*/*I*_D_^2^ mean value increases as the area gets larger and as a result *N*_T_, *α*_H_ get also higher as shown in Table 1. Table S1† clearly demonstrates that variances of *N*_tr_, *α*_H_ respectively, increase more strongly than their mean values as the devices' area increases. The latter can justify the boost of *N*_tcoeff_ parameter and the reduction of *N*_*α*_H__ as the dimensions get larger. The reason for these variations of the statistical LFN model parameters which occur as a physical consequence of the variations of the LFN mean value model ones, could be the critical inhomogeneities of GFET technologies, as it has already been stated before.

Standard deviation of natural logarithm *σ*(ln[*WLS*_*I*_D__*f*/*I*_D_^2^]) of normalized LFN is shown in Fig. 6 and some remarkable conclusions can be extracted. The above quantity can be used as a figure of merit for LFN variability comparisons between the GFET technology in this work and CMOS ones^35–37,40^ It can be concluded that the range of values between 0.1–1 for all the transistors under test as depicted in Fig. 6 are similar with the results obtained from CMOS devices with similar dimensions, indicating a decent performance for the GFETs under study.^35–37,40^ Another crucial observation is the weak bias dependence of *σ*(ln[*WLS*_*I*_D__*f*/*I*_D_^2^]) data with a rather smooth fluctuation near the CNP and a slight decrease away from the CNP, which is remarkably captured by the proposed model.

Despite the fact that the proposed *WLS*_*I*_D__*f*/*I*_D_^2^ variance model focuses on the bias dependence, its scaling with the area is also crucial. Δ*N* and Δ*μ WLS*_*I*_D__*f*/*I*_D_^2^ variance models follow a ∼1/(*WL*) trend as it is clear from eqn (9) and (13) and as a result total LFN variance expression in eqn (14) behaves similarly. *S*_*I*_D__*f*/*I*_D_^2^ mean value model presents the same dependency.^53–55^ In addition, *σ*(ln[*WLS*_*I*_D__*f*/*I*_D_^2^]) also follows an ∼√1/(*WL*) trend due to the large device dimensions.^25,27,35–37,40^ However, due to inhomogeneities of the GFET technologies in general, extracted parameters both of the LFN mean value (*N*_T_, *α*_H_) and variance (*N*_tcoeff_, *N*_*α*_H__) models, differ from device to device.

## Conclusions

This work investigates thoroughly the bias dependence of LFN variability in large area SG-SL GFETs. To our knowledge, this is the first time that such a study that combines both the analysis of statistical LFN data for graphene devices and the extraction of a physics-based LFN variance model is presented. More specifically, an analytical compact model based on carrier number Δ*N* and mobility fluctuation Δ*μ* effects has been proposed and implemented for circuit simulators. The development of such an effective tool is critical for the boost of graphene circuit design, where LFN variability should be accurately predicted to prevent performance deterioration in certain applications. The latter is one of the most significant novelties of this work. Besides, it is experimentally proven that LFN variability does not count on the variability of *IV* quantities such as *V*_CNP_, *I*_D_ but it is directly linked to the number of traps *N*_tr_ and Hooge parameter *α*_H_ variations regarding Δ*N* and Δ*μ* mechanisms respectively. The derived compact model precisely covers the measured LFN variance over the whole range of operation, from strong conduction to the CNP at both p- and n-type regimes. Δ*N* and Δ*μ* LFN variance models exhibit a similar bias dependence with the corresponding Δ*N* and Δ*μ* mean value ones. Thus, Δ*N* effect accounts for the M-shape of LFN variance similarly as it is known to cause an M-shape for its mean value while Δ*μ* provides a Λ-shape in the bias dependence of LFN variance, which contributes significantly at the CNP, analogously to the Δ*μ* contribution to LFN mean value. It is also worth mentioning that variances of both Δ*N* and Δ*μ* mechanisms must be computed locally in the channel in order to guarantee that each parameter's (*N*_tr_, *α*_H_) deviation at any position in the channel will have an individual impact on LFN variability. A simpler variance model with a ∼(*g*_m_/*I*_D_)^4^ shape is also extracted based on a uniform channel approximation and shown for comparison reasons. This approach based on the well-known ∼(*g*_m_/*I*_D_)^2^ model of the Δ*N* contribution to LFN mean value fails to accurately predict the LFN variance near the CNP.

*N*_T_, (or *N*_tr_ consequently) and *α*_H_ parameters of the LFN model mean value are also used in LFN variance model together with the newly defined *N*_tcoeff_ and *N*_*α*_H__ parameters which are extracted from statistical LFN data. In silicon-oxide transistors, *N*_tr_ follows a Poisson distribution and thus, *N*_tcoeff_ is close to unity but this is not the case in the devices under test. The latter is experimentally shown by extracting LFN mean value model parameters for every measured sample and then by calculating their variance and mean value.

Regarding geometrical scaling, while both *S*_*I*_D__*f*/*I*_D_^2^ mean value and *WLS*_*I*_D__*f*/*I*_D_^2^ variance models predict a ∼1/(*WL*) behavior, the corresponding data do not follow this trend. In fact, the extracted LFN parameters are not identical for the three GFETs under test despite the fact that they are devices of the same technology. On the contrary, LFN mean value parameters increase with device area causing also a large deviation in LFN variance parameters. This device to device parameters' deviation could be justified due to increased inhomogeneities observed on recently developed GFET technologies.

Eqn (15) expresses the standard deviation of natural logarithm of *WLS*_*I*_D__*f*/*I*_D_^2^ – *σ*(ln[*WLS*_*I*_D__*f*/*I*_D_^2^]) – which is taken into consideration and examined due to log-normal distribution of LFN data and it might be a reliable tool in order to compare LFN variance for different types of transistors. This study represents the first reported efforts to understand LFN variability in GFETs. The derived results contribute to the thorough understanding of the nature of charge traps statistics in solution-gated devices and they also provide the tools to quantify and predict LFN variability in SL GFETs. This framework is considered critical for upscaling the production of graphene electronics from research labs into larger-scale dedicated fabrication facilities.

## Experimental data

### Electrical characterization of LFN

To measure the DC transfer curves and the LFN spectra accurately, the drain to source current was pre-amplified in a first amplification stage with a gain of 10^4^. The signal was then high pass filtered, thus canceling its low frequency (*i.e.* DC level) components. The resulting signal was further amplified and low pass (anti-aliasing) filtered in a second stage with a 10^2^ gain. The signals were digitalized using a NI DAQ Card in all characterization procedures. To extract the power spectral density, the drain to source current was measured under different gate bias conditions for 10 s at each point.

### Fabrication of SG GFETs

Arrays of SG-SL GFETs were fabricated on a 10 μm thick polyimide (PI-2611, HD MicroSystems) film spin coated on a Si/SiO2 4′′ wafer and baked at 350 °C. A first metal layer (10 nm Ti/100 nm Au) was deposited by electron-beam vapour and then structured by a lift-off process. Afterwards, the graphene grown by chemical vapour deposition on Cu was transferred (process done by Graphenea s.a.). Graphene was then patterned by oxygen plasma (50 sccm, 300 W for 1 min) in a reactive ion etching (RIE) after protecting the graphene in the channel region with HIPR 6512 (FujiFilm) positive photoresist. After the graphene etching, a second metal layer was patterned on the contacts following the same procedure as for the first layer. The lift-off was followed by an annealing in ultra-high vacuum consisting on a temperature ramp from room temperature to 300 °C. Subsequently, the transistors were insulated with a 3 μm-thick photodefinable SU-8 epoxy photoresist (SU-8 2005 Microchem), keeping uncovered the active area of the transistors channel and the contacting pads. The polyimide substrate was structured in a reactive ion etching process using a thick AZ9260 positive photoresist (Clariant) layer as an etching mask. The neural probes were then peeled off from the wafer and placed in a zero-insertion force connector to be interfaced with our custom electronic instrumentation. Finally, the devices were rinsed for 2 minutes in ethanol to eliminate remaining resist residues on the graphene channel.

### Raman characterization of graphene after transfer

A SLG sample was transfer onto a SiO_2_ wafer following the same process as detailed for the fabrication of GFETs. The Raman spectra at 400 equally spaced points were acquired on the graphene sample, within an area of 20 μm × 20 μm. A Witec spectrometer in backscattering configuration, using a 600 gr per nm grating was used. A 488 nm wavelength laser (2.5 mW power) was focused on the sample with a 50× objective. The peak intensity for the D and G bands was measured after background subtraction.

## Data availability

The data that support the findings of this study are available from Ramon Garcia Cortadella. Please, address your requests to ramon.garcia@icn2.cat.

## Conflicts of interest

There are no conflicts to declare.

## Supplementary Material

NA-002-D0NA00632G-s001
