# Supplementary material for: Bias dependent variability of low-frequency noise in single-layer graphene FETs
Source: Nanoscale Adv. 2020 Oct 26;2(11):5450–60. doi: 10.1039/d0na00632g (PMC9418965; doi:10.1039/d0na00632g)
Supplement: NA-002-D0NA00632G-s001 [file NA-002-D0NA00632G-s001.pdf]

Electronic Supplementary Material (ESI) for Nanoscale Advances.
This journal is © The Royal Society of Chemistry 2020

Supplementary Information for:

Bias Dependent Variability of Low-Frequency Noise in Single-Layer Graphene FETs

Nikolaos Mavredakis, Ramon Garcia Cortadella, Xavi Illa, Nathan Schaefer, Andrea Bonaccini Calia, Anton Guimerà-Brunet, Jose Antonio Garrido and David Jiménez

A. Supplementary Information: Definitions and equations for IV-LFN model

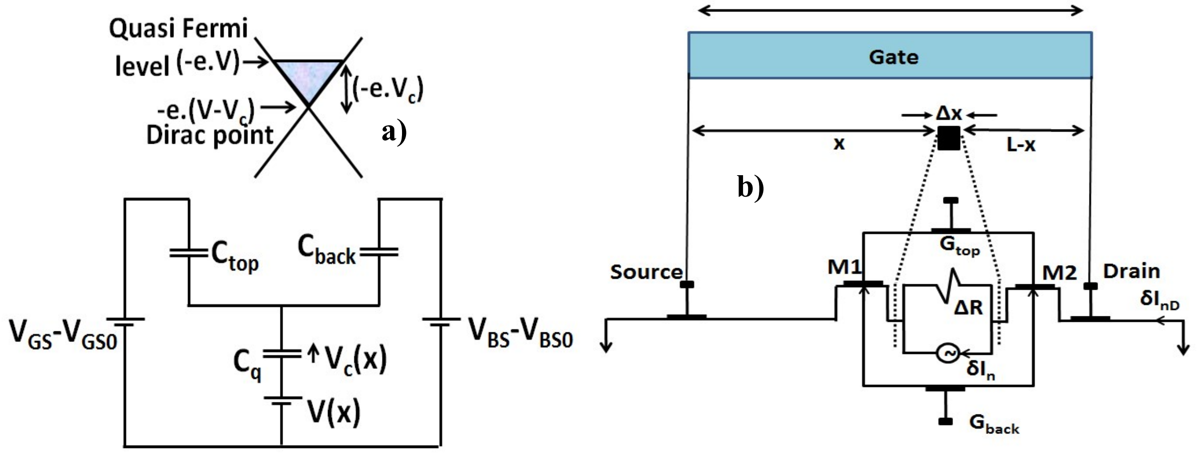

Fig. S1. a) Energy dispersion diagram of GFET (top) and its capacitive circuit (bottom) are shown. b) The equivalent circuit for a local current noise contribution to the total noise is illustrated. Each noise-generating slice of the channel is connected to two noiseless GFETs, M1 and M2 respectively.

Fig. S1a depicts the equivalent capacitive circuit of the CV-IV chemical potential based model[56-58] where quantum capacitance (C_q) is the derivative of graphene charge Q_{gr} and chemical potential $V_c(x)$ corresponds to the voltage drop across C_q at channel position x. A linear relationship is considered between C_q and V_c. ($C_q=k|\ V_c\ |$) where $k=2e^3/(\pi h^2 u^2 f)$ [56-58] where u_f is the Fermi velocity ($=10^6\ m/s$), h the reduced Planck constant ($=1{,}05\cdot 10^{-34}\ J.s$). $V_c(x)$ equals to the potential difference between the quasi-Fermi level and the potential at the CNP, as illustrated in the energy dispersion relation scheme of graphene in the top drawing of Fig. S1a where $V_c(0)=V_{cs}$ and $V_c(L)=V_{cd}$ at the Source ($x=0$) and Drain ($x=L$) end, respectively. Top and back gate source voltage overdrives are represented as: V_{GS}-V_{GS0}, V_{BS}-V_{BS0} while top and back gate capacitances as: C_{top} and C_{back}. In the GFETs of the present study, only top gate voltage is applied and thus C_{back} is considered negligible. $V(x)$ is the graphene channel quasi-

Fermi potential at position x, which equals to zero at the Source and V_{DS} at the Drain end respectively. Bias dependent term g_{vc} defined in the main manuscript is calculated as[56-58]:

$$g_{V_c} = \left[g\left(V_c\right) \right]_{V_{cs}}^{V_{cd}} + \frac{\alpha V_{DS}}{k} = \frac{V_{cs}^3 - V_{cd}^3}{3} + \frac{k}{4C}\left[\operatorname{sgn}\left(V_{cd}\right) V_{cd}^4 - \operatorname{sgn}\left(V_{cs}\right) V_{cs}^4 \right] + \frac{\alpha V_{DS}}{k} \quad \text{(S1)}$$

while the drain current expression is[56-58]:

$$I_D = \frac{\mu W k}{2L} g_{V_c} \quad \text{(S2)}$$

Graphene charge is given by[56-58]:

$$Q_{gr} = \frac{k}{2}\left(V_c^2 + \alpha / k \right) \quad \text{(S3)}$$

and chemical potential at Source and Drain as[56-58]:

$$V_{cs,d} = \frac{C - \sqrt{C^2 \pm 2k\left[C_{top}\left(V_{Gtop} - V_{Gtop0} - V_{s,d} \right) + C_{back}\left(V_{Gback} - V_{Gback0} - V_{s,d} \right) \right]}}{\pm k} \quad \text{(S4)}$$

As thoroughly explained in the main manuscript, the integration of local LFN noise sources across the channel leads to the calculation of the total LFN PSD and its variance. In order to obtain an analytical compact solution based on the recently established chemical potential based IV model[56-58], a change of integral variable occurs from length x to chemical potential V_c:

$$\frac{dx}{dV_c} = \frac{-\mu W Q_{gr}}{I_D} \frac{C_q + C}{C} \quad \text{(S5)}$$

The fluctuations producing LFN are always slight and as a result, the analysis of the propagation of the noise sources to the voltages or currents at the contact terminals reduces to linear analysis. Therefore, the principle of superposition can be used for adding the effects of the local noise sources along the channel[33]. These local fluctuations can be modeled by adding a random local current noise source δI_n with a PSD $S\delta I^2_n$ as shown in the equivalent noise subcircuit[53-54] in Fig. S1b. The local fluctuations propagate to the terminals resulting in fluctuations of the voltages and currents around the DC operating point.

B. **Supplementary Information: *CDF and V_{GEFF}-$WLS_{ID}f/I_D^2$* variance relation for *W/L=20 µm/20 µm, 50 µm/50 µm* GFETs.**

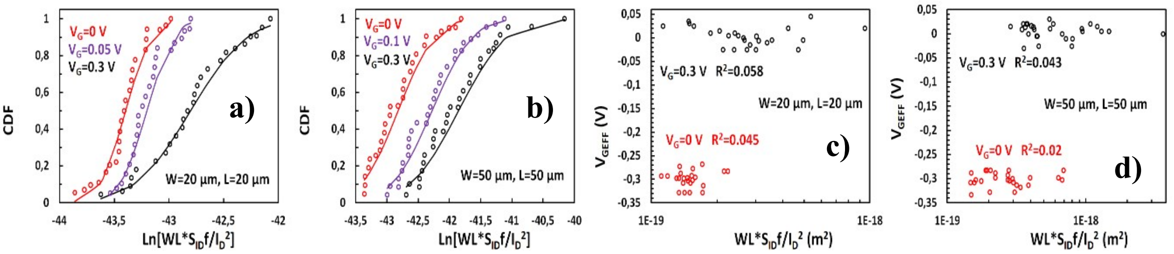

Fig. S2. Cumulative distribution function (CDF) of natural logarithm of normalized LFN $Ln(WLS_{ID}f/I_D^2)$, referred to *1 Hz*, for GFET with a) *W/L=20 µm/20 µm* and b) *W/L=50 µm/50 µm*, shows a log-normal distribution. Markers: extracted CDF, solid lines: theoretical CDF of normal distribution of $Ln(WLS_{ID}f/I_D^2)$. c) Variability of $WLS_{ID}f/I_D^2$, referred to *1 Hz*, is much higher and uncorrelated with variability of V_{GEFF} for GFET with c) *W/L=20 µm/20 µm* and d) *W/L=50 µm/50 µm*.

Fig. S2a and S2b depict the CDF of the natural logarithm of $WLS_{ID}f/I_D^2$ for the *20 µm/20 µm*, *50 µm/50 µm* GFETs respectively, at three different V_{GEFF} values clearly indicating the log-normal distribution of their $WLS_{ID}f/I_D^2$ data. These data variability is also shown vs. V_{GEFF} variability in Fig. S2c and S2d for the same devices and it is apparent that these two quantities are not correlated which means that $WLS_{ID}f/I_D^2$ variance is mostly related with the mechanisms that produce LFN, as proved in the main manuscript.

C. **Supplementary Information: Complete LFN variance model including Velocity Saturation effect on *ΔN, Δμ* mechanisms**

In ref. 54, the effect of Velocity Saturation (VS) on LFN mean value model and the way it reduces *ΔN* and *Δμ* contributions under high electric fields and short channel dimensions is analyzed in detail. In the present section, a similar analysis will be performed regarding VS effect on *ΔN* and *Δμ* LFN variance. The methodology followed is identical to the one used in ref. 54 where eqn (2) of the ref. 54 is applied in order to change the integral variable from x to V_c instead of eqn (S5) mentioned above. As a result, additional *ΔN*, *Δμ* VS induced LFN variance terms will be added to the long channel terms derived in the main manuscript. More particularly, eqns (8-9, 12-13) of the main manuscript regarding *ΔN*, *Δμ* LFN variance

contributors, respectively, correspond to the long channel terms named as *Var[ΔNA]*, *Var[ΔμA]* in this section. Taking into consideration the effective length L_{eff} which accounts for the degradation of I_D because of VS effect, the following expressions are derived:

$$Var\left[\frac{S_{I_D}}{I_D^2}\bigg|_{\Delta NA} WLf\right] = \frac{L^2}{L_{eff}^3}\frac{16kT\lambda N_{tcoeff} N_T e^4}{W g_{vc} C}\int_{V_{cd}}^{V_{cs}} \frac{V_c^4}{\left(V_c^2 + a/k\right)^3 \left(k\left|V_c\right| + C\right)^3} dV_c \tag{S6}$$

$$Var\left[\frac{S_{I_D}}{I_D^2}\bigg|_{\Delta \mu A} WLf\right] = \frac{L^2}{L_{eff}^3}\frac{4e^2\alpha_H}{W N_{\alpha_H} g_{vc} C k^2}\int_{V_{cd}}^{V_{cs}} \frac{k\left|V_c\right| + C}{V_c^2 + a/k} dV_c \tag{S7}$$

If eqns (S6-S7) are analytically solved, they result in eqns (9, 13) of the main manuscript, respectively, with the difference of the contribution of L_{eff} which is given by eqns (A10-A11) of the Supporting Information of ref. 54. Thus, *1/L* term in eqns (8-9, 12-13) of the main manuscript is replaced with L^2/L^3_{eff} term in eqns (S6-S7), respectively, where in case of a long L ($L_{eff} \approx L$), these two terms are equivalent ($1/L \approx L^2/L^3_{eff}$). Constant terms of eqns (S6-S7) are defined as: $A1Var = \frac{16kT\lambda N_{tcoeff} e^4}{WC}$ and $A2Var = \frac{4e^2\alpha_H}{W N_{\alpha H} C k^2}$. The contribution of the 2nd term of eqn (2) of ref. 54 results in *Var[ΔNB]*, *Var[ΔμB]* terms of the LFN variance model which are fully equivalent to the *ΔNB*, *ΔμB* ones of the LFN mean-value model in ref. 54. In more detail:

$$Var\left[\frac{S_{I_D}}{I_D^2}\bigg|_{\Delta NB} WLf\right] = \frac{L^2}{L_{eff}^4} A1Var\mu \int_{V_{cd}}^{V_{cs}} \frac{V_c^4 \left(k\left|V_c\right| + 2C\right)}{u_{sat}\left(V_c^2 + a/k\right)^4 \left(k\left|V_c\right| + C\right)^4} dV_c \tag{S8}$$

$$Var\left[\frac{S_{I_D}}{I_D^2}\bigg|_{\Delta \mu B} WLf\right] = \frac{L^2}{L_{eff}^4} A2Var\mu \int_{V_{cd}}^{V_{cs}} \frac{k\left|V_c\right| + 2C}{u_{usat}\left(V_c^2 + a/k\right)^2} dV_c \tag{S9}$$

According to eqn (4) of ref. 54, $u_{sat}=2u_f/\pi=S$ is constant near CNP for $V_c \leq V_{ccrit}$ where V_{ccrit} is a critical value of V_c[54] while for $V_c \geq V_{ccrit}$, $u_{sat}=N/\surd(V^2_c+a/k)$ is inversely proportional to V_c; where $N= h\Omega u_f/e$ and $h\Omega$ is phonon energy used as an IV model parameter[54-55] which is considerable only at high electric field region. Thus, eqns (S8-S9) become:

$$Var\left[\frac{S_{I_D}}{I_D^2}\bigg|_{\Delta NB} WLf\right]_{V_c \leq V_{ccrit}} = \frac{L^2}{L_{eff}^4}\frac{A1Var\mu}{S} \int_{V_{cd}}^{V_{cs}} \frac{V_c^4 \left(k\left|V_c\right| + 2C\right)}{\left(V_c^2 + a/k\right)^4 \left(k\left|V_c\right| + C\right)^4} dV_c \tag{S8a}$$

$$Var\left[\frac{S_{I_D}}{I_D^2}\bigg|_{\Delta \mathrm{N}B} WLf\right]_{V_c \geq V_{ccrit}} = \frac{L^2}{L_{eff}^4}\frac{A1Var\mu}{N}\int_{V_{cd}}^{V_{cs}} \frac{V_c^4\left(k\left|V_c\right|+2C\right)\sqrt{V_c^2+a/k}}{\left(V_c^2+a/k\right)^4\left(k\left|V_c\right|+C\right)^4}dV_c \qquad \text{(S8b)}$$

$$Var\left[\frac{S_{I_D}}{I_D^2}\bigg|_{\Delta \mu B} WLf\right]_{V_c \leq V_{ccrit}} = \frac{L^2}{L_{eff}^4}\frac{A2Var\mu}{S}\int_{V_{cd}}^{V_{cs}} \frac{k\left|V_c\right|+2C}{\left(V_c^2+a/k\right)^2}dV_c \qquad \text{(S9a)}$$

$$Var\left[\frac{S_{I_D}}{I_D^2}\bigg|_{\Delta \mu B} WLf\right]_{V_c \geq V_{ccrit}} = \frac{L^2}{L_{eff}^4}\frac{A2Var\mu}{N}\int_{V_{cd}}^{V_{cs}} \frac{k\left|V_c\right|+2C}{\left(V_c^2+a/k\right)^{3/2}}dV_c \qquad \text{(S9b)}$$

which can analytically be solved as:

$$Var\left[\frac{S_{I_D}}{I_D^2}\bigg|_{\Delta \mathrm{N}B} WLf\right]_{V_c \leq V_{ccrit}} = \frac{L^2}{L_{eff}^4}\frac{A1Var\mu}{S}\frac{1}{24\left(C^2+ak\right)^7}$$

$$\Bigg[\mp\frac{8C^5k^3\left(C^2+ak\right)^3}{\left(C\pm kV_c\right)^3}\mp\frac{4ak^2\left(C^2+ak\right)^3\left(a^3k^2\mp 2C^5V_c+2a^2Ck\left(C\pm kV_c\right)\right)\pm aC^3\left(8kV_c\mp 7C\right)}{\left(a+kV_c^2\right)^3}$$
$$\pm\frac{12k^3\left(C^2+ak\right)^2\left(-5C^6+3aC^4k\right)}{\left(C\pm kV_c\right)^2}\mp\frac{48C^3k^3\left(C^2+ak\right)\left(7C^4-12aC^2k+a^2k^2\right)}{C\pm kV_c}-$$
$$\frac{2Ck^2\left(C^2+ak\right)^2\left(7C^6V_c\pm 3aC^4\left(\mp 23kV_c+14C\right)+5a^2C^2k\left(5kV_c\mp 12C\right)+a^3k^2\left(5kV_c\mp 6C\right)\right)}{\left(a+kV_c^2\right)^2}-$$
$$\frac{3Ck^2\left(C^2+ak\right)\left(\pm C^8V_c-4a^2C^4k\left(\mp 53kV_c+48C\right)+2aC^6\left(\mp 41kV_c+14C\right)+a^4k^3\left(\mp 5kV_c+8C\right)\right)+6a^3C^2k^2\left(\mp 5kV_c+18C\right)}{a\left(a+kV_c^2\right)}+$$
$$\frac{3Ck^{3/2}\left(C^{10}+47aC^8k-574a^2C^6k^2+630a^3C^4k^3-35a^4C^2k^4-5a^5k^5\right)a\tan\left(\sqrt{\frac{k}{a}}V_c\right)}{a^{3/2}}\pm$$
$$24C^2k^3\left(15C^6-49aC^4k+17\left(aCk\right)^2+\left(ak\right)^3\right)\ln\left(\frac{\left(C\pm kV_c\right)^2}{a+kV_c^2}\right)\Bigg]_{V_{cd}}^{V_{cs}}$$

(S10a)

*$\pm, \mp$: Top sign refers to Vc>0 and bottom sign to Vc <0.

$$Var\left[\frac{S_{I_D}}{I_D^2}\bigg|_{\Delta NB} WLf\right]_{V_c \geq V_{ccrit}} = \frac{L^2}{L_{eff}^4}\frac{A1Var\mu}{N}\frac{1}{30}k^2$$

$$\left[\frac{\sqrt{V_c^2+a/k}}{\left(C^2+ak\right)^6}\left(\begin{array}{l} \mp\frac{10C^5k\left(C^2+ak\right)^2}{\left(C\pm kV_c\right)^3}\mp\frac{5C^4k\left(14C^2-9ak\right)\left(C^2+ak\right)}{\left(C\pm kV_c\right)^2}\mp\frac{5C^3k\left(68C^4-133aC^2k+12a^2k^2\right)}{C\pm kV_c}- \\ \frac{4C\left(C^2+ak\right)\left(6C^6V_c\pm aC^4\left(35C\mp 58kV_c\right)+10a^2C^2k\left(2kV_c\mp 5C\right)+a^3k^2\left(4kV_c\mp 5C\right)\right)}{\left(a+kV_c^2\right)^2}\mp \\ \frac{6a\left(C^2+ak\right)^2\left(a^3k^2\mp 2C^5V_c+2a^2Ck\left(C\pm kV_c\right)\pm aC^3\left(8kV_c\mp 7C\right)\right)}{\left(a+kV_c^2\right)^3}\pm \\ \frac{2C\left(\begin{array}{l}\pm 6C^8V_c+aC^6\left(105C\mp 332kV_c\right)+3\left(aCk\right)^2\left(135C\mp 32kV_c\right)+2a^4k^3\left(15C\mp 8kV_c\right)\pm \\ 2a^2C^4k\left(391kV_c\mp 360C\right)\end{array}\right)}{a\left(a+kV_c^2\right)} \end{array}\right)\pm \right.$$

$$\left.\frac{15C^2\left(32C^6-142aC^4k+61\left(aCk\right)^2+4\left(ak\right)^3\right)}{\left(C^2+ak\right)^{13/2}}\ln\left(\frac{C\pm kV_c}{a\mp CV_c+\sqrt{\left(C^2+ak\right)\left(V_c^2+a/k\right)}}\right)\right]_{V_{cd}}^{V_{cs}}$$

(S10b)

$$Var\left[\frac{S_{I_D}}{I_D^2}\bigg|_{\Delta\mu B} WLf\right]_{V_c \leq V_{ccrit}} = \frac{L^2}{L_{eff}^4}\frac{A2Var\mu}{S}k^2\left[\frac{2CV_c\mp a}{2a\left(a+kV_c^2\right)}+\frac{Ca\tan\left(\sqrt{\frac{k}{a}}V_c\right)}{a^{3/2}\sqrt{k}}\right]_{V_{cd}}^{V_{cs}} \quad \text{(S11a)}$$

$$Var\left[\frac{S_{I_D}}{I_D^2}\bigg|_{\Delta\mu B} WLf\right]_{V_c \geq V_{ccrit}} = \frac{L^2}{L_{eff}^4}\frac{A2Var\mu}{N}k^2\left[\frac{2CV_c\mp a}{a\left(a+kV_c^2\right)}\sqrt{\frac{V_c^2+a/k}{k}}\right]_{V_{cd}}^{V_{cs}} \quad \text{(S11b)}$$

Finally we get *Var[ΔN]= Var[ΔNA]+Var[ΔNB]*, *Var[Δμ]= Var[ΔμA]+Var[ΔμB]* and *Var[Total]= Var[ΔN]+Var[Δμ]* (S12)

Eqn (S12) is equivalent to eqn (14) of the main manuscript. Despite the fact that in the LFN mean value model, the VS related terms *ΔNB*, *ΔμB* are subtracted from *ΔNA, ΔNB* respectively[54-55], variances are always summed since the variance of the difference of two random variables equals to the sum of the variances of these variables.

The graphical representation of LFN variance contributors derived above (eqns S6-S12) is

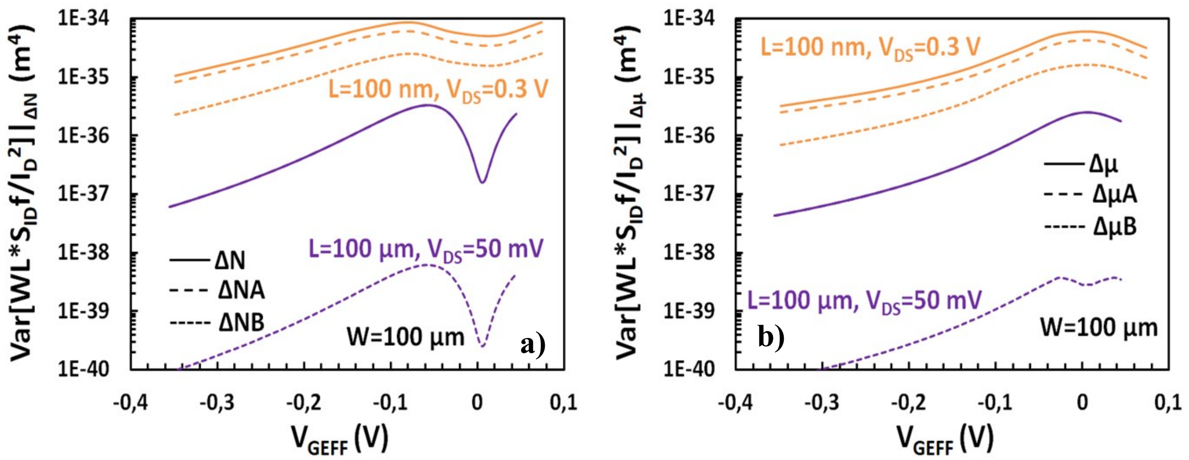

Fig. S3. Variance of normalized LFN $Var[WLS_{\delta In}f/I_D^2]$, referred to $1\ Hz$, vs. top gate voltage overdrive V_{GEFF}, for a) ΔN effect and b) $\Delta\mu$ effect for a GFET with $W=100\ \mu m$ at two different L and V_{DS} cases; i) $L=100\ \mu m$, $V_{DS}=50\ mV$ (purple) and ii) $L=100\ nm$, $V_{DS}=0.3\ V$ (orange). Dashed lines represent ΔNA, ΔNB contributors of ΔN effect in (a) and $\Delta\mu A$, $\Delta\mu B$ contributors of $\Delta\mu$ effect in (b) while solid lines the total ΔN, $\Delta\mu$ LFN variance mechanisms.

shown in Fig. S3 for both low ($L=100\ \mu m$, $V_{DS}=50\ mV$) and high electric field ($L=100\ nm$, $V_{DS}=0.3\ V$) in order to investigate the contribution of VS related terms (ΔNB, $\Delta\mu B$) to total variance. Fig. S3a represents ΔN effect and its contributors (ΔNA, ΔNB) while Fig. S3b $\Delta\mu$ effect and its contributors ($\Delta\mu A$, $\Delta\mu B$). It can be concluded that under long channel and low V_{DS} conditions which is the case of the measured GFETs at the present work, the effect of VS on LFN variance is negligible. The testing of the model at higher drain voltage region for quite short channel length (same parameters from Table 1 of the main manuscript and $h\Omega=18\ meV$) reveals a contribution of VS effect to LFN variance which results in the increase of the later. Similar conclusions had been extracted for the effect of VS on LFN mean value model[54-55] with the difference that in that case VS effect reduced total LFN under high electric fields.

D. Supplementary Information: Mathematical proof of eqn (6) of the main manuscript

In the case of ΔN effect, uncorrelated local noise sources inside the integral of eqn (5) of the main manuscript can be expressed as $f(N_{tr})=\Lambda(x)N_{tr}$ where $\Lambda(x)=(eC_q(x))^2[LQ_{gr}(x)\ (C_q(x)+C)]^{-2}W^{-1}$ with Q_{gr}, C_q functions of x. If eqn (3) of the main manuscript is considered, eqn (6) of the main manuscript can be analyzed as:

$$Var\left[\int_0^L f(N_{tr})dx\right] = Var\left[\int_0^L \Lambda(x)N_{tr}dx\right] = \left[\int_0^L Var\left[\Lambda(x)N_{tr}dx\right]\right] = \int_0^L Var\left[N_{tr}\right]\left(\Lambda(x)dx\right)^2$$
$$= \int_0^L\int_0^L Var\left[N_{tr}\right]\Lambda^2(x)dxdy = L\int_0^L Var\left[N_{tr}\right]\Lambda^2(x)dx = L\int_0^L Var\left[f(N_{tr})\right]dx \tag{S13}$$

Variance can get into the sum since local noise sources are considered independent while squared integral variable leads to a double integral notation. Since $\Lambda^2(x)$ is function only of one variable (x), the double integral turns into a single integral multiplied with L^*. Eqn (S13) proves the validity of eqn (6) of the main manuscript. The same procedure can be applied for $\Delta\mu$ effect.

$$* \quad A = \int_0^L \Lambda^2(\chi)(dx^2) \Leftrightarrow \frac{d^2A}{dx^2} = \frac{d\,dA}{dxdx} = \Lambda^2(\chi) \Leftrightarrow \frac{dA}{dx} = \int_0^L \Lambda^2(\chi)dx =$$
$$M(L) - M(0) \Leftrightarrow A = \int_0^L (M(L) - M(0))dx = L(M(L) - M(0)) = L\int_0^L \Lambda^2(\chi)dx, \quad M(x) = \int \Lambda^2(x)dx$$

E. Supplementary Information: ~$(g_m/I_D)^4$ LFN Variance Model

In Fig. 5a of the main manuscript, a simple ~$(g_m/I_D)^4$ $WLS_{ID}f/I_D^2$ variance model is shown which is valid only away the CNP where channel is uniform. It is based on a very common approximation for ΔN LFN mean value modeling which predicts a ~$(g_m/I_D)^2$ dependence[26, 29, 31]. The way this variance model is derived is shown in analytical steps in the present section. According to eqn (A23) in the Supporting Information of ref. 53, current fluctuation ΔI_D equals to:

$$\Delta I_D = \frac{1}{LC} g_m e \int_0^L \Delta N_t dx \tag{S14}$$

which in terms of $WLS_{ID}f/I_D^2$ results in:

$$\left.\frac{S_{I_D}}{I_D^2}\right|_{\Delta \mathrm{N}} WLf = \left(\frac{g_m}{I_D}\right)^2 \frac{e^2 WLKT\lambda N_T}{WLC^2} \tag{S15}$$

Since the channel is considered uniform in this approach, no quantity remains inside the integral[53] and thus, there is no point of calculating the local variance. If total variance of eqn (S15) is calculated:

$$
\begin{aligned}
&Var\left[\frac{S_{I_D}}{I_D^2}\bigg|_{\Delta \mathrm{N}} WLf\right] = Var\left[\left(\frac{g_m}{I_D}\right)^2 \frac{e^2 N_{tr}}{WLC^2}\right] = \\
&\left(\frac{g_m}{I_D}\right)^4 \frac{e^4}{(WL)^2 C^4} Var\left[N_{tr}\right] = \left(\frac{g_m}{I_D}\right)^4 \frac{KTe^4 \lambda N_{tcoeff} N_T}{WLC^4}
\end{aligned}
\qquad \text{(S16)}
$$

F. Supplementary Information: Analysis of LFN variance locally in the channel

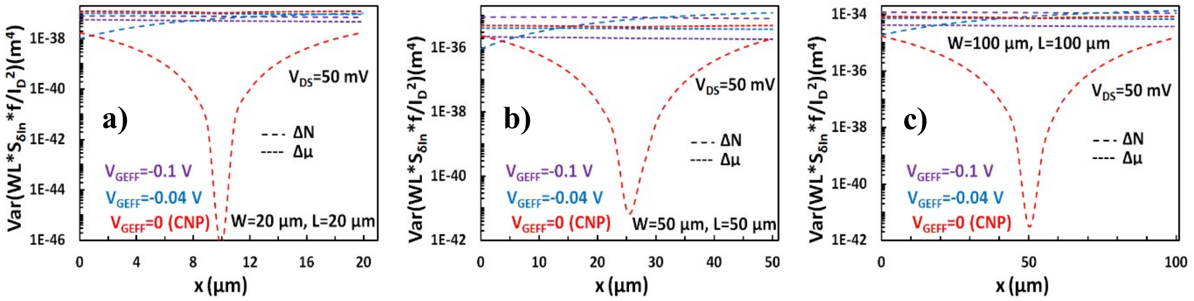

Fig. S4. Local variance of normalized LFN $Var[WLS_{\delta In}f/I_D^2]$, referred to *1 Hz*, vs. channel position x, for GFETs with a) W/L=*20 μm/20 μm*, b) *50 μm/50 μm* and c) *100 μm/100 μm*. ΔN, $\Delta\mu$ contributions are shown at three different V_{GEFF} values away, close and at the CNP (V_{GEFF}=-*0.1*, -*0.04* and *0 V*).

In Fig. S4, the local LFN variance is illustrated vs. channel position x for all the GFETs under study; a) W/L=*20 μm /20 μm*, b) *50 μm/50 μm* and c) *100 μm/100 μm*. Both LFN mechanisms (ΔN–inside the integral of eqn (8) in the main manuscript, $\Delta\mu$–inside the integral of eqn (12) in the main manuscript) are depicted far from, close to and at the CNP in the p-type region. What can be noticed is that ΔN local LFN variance near the CNP is mainly determined by the Source and Drain areas while at the middle of the channel where the CNP occurs under low V_{DS}, it presents a deep minimum and becomes trivial. Away from the CNP it is constant along the channel due to its homogeneity. Regarding $\Delta\mu$ local LFN variance, it remains continual both near and away the CNP. The above dependencies of LFN local variances resemble the LFN local mean values[53]. In more detail, the dip observed at the CNP for the ΔN local LFN variance

is probably caused by the residual charge ρ_0 as the latter is known to cause a similar shape for the ΔN local LFN mean value[53]. Both $\Delta\mu$ local LFN variance and mean value are not very sensitive to the residual charge.

G. Supplementary Information: Extraction of N_{tr}, α_H, N_{tcoeff} and $N_{\alpha H}$ parameters

The N_{tcoeff}, $N_{\alpha H}$ statistical LFN models parameters are extracted from LFN variance data in Fig. 5 of the main manuscript. Regarding N_{tcoeff}, it is much greater than unity for all GFETs which means that the Poisson distribution that is the case for trap statistics in silicon-oxide transistors, is not valid in this study probably because of a different nature of traps due to electrolyte-graphene interface[43, 60-63]. To investigate deeper the latter, LFN mean value model parameters (N_T, α_H) are extracted for every available measured sample for each one of the three GFETs of this work; N_{tr} can be easily calculated from N_T. Fig. S5 presents the $WLS_{ID}f/I_D^2$ LFN mean value data from 5 random samples for a) $W/L= 20\ \mu m/20\ \mu m$, b) $50\ \mu m/50\ \mu m$ and c) $100\ \mu m/100\ \mu m$ GFETs vs. V_{GEFF}. Measurements are shown with markers while the models are also derived for every sample and shown with solid lines. The values of the extratced N_{tr}, α_H parameters for every sample are shown in Table S1 while their variance and ln-mean value are also estimated. As it was indicated in the main manuscript, $N_{tcoeff}=Var[N_{tr}]/E[N_{tr}]$ and $N\alpha_H=E[\alpha_H](Var[\alpha_H]WL)^{-1}$, thus the LFN variance model parameters can be derived. Astonishingly, these calculated parameters are exact dublicates of the values in Table 1 of the main manuscript which were extracted from LFN variance data of Fig. 5 of the main manuscript and resulted in the remarkable accuracy of the model.

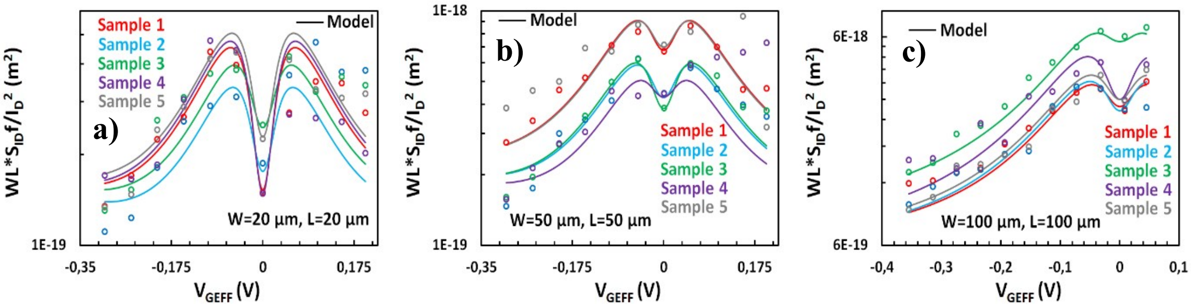

Fig. S5. Normalized LFN $WLS_{ID}f/I_D^2$, referred to $1\ Hz$, vs. top gate voltage overdrive V_{GEFF}, for

GFETs with a) *W/L=20 µm/20 µm*, b) *50 µm/50 µm* and c) *100 µm/100 µm*. Measured noise from 5 different samples: open circle markers, model for 5 different samples: lines. (sample 1: red, sample 2: blue, sample 3: green, sample 4: purple, sample 5: grey).

TABLE S1

	**W/L=20 *µm*/20 *µm***		**W/L=50 *µm*/50 *µm***		**W/L=100 *µm*/100 *µm***	
	**N_{tr}=WLKTλN_T**	**α_H**	**N_{tr}=WLKTλN_T**	**α_H**	**N_{tr}=WLKTλN_T**	**α_H**
**1**	$2.925416.10^{4}$	$7.15.10^{-4}$	$3.270822.10^{5}$	$0.833.10^{-3}$	$5.0411456.10^{6}$	$1.75.10^{-2}$
**2**	$1.331897.10^{4}$	$10.4.10^{-4}$	$2.559351.10^{5}$	$1.943.10^{-3}$	$2.3266826.10^{6}$	$0.9375.10^{-2}$
**3**	$3.329741.10^{4}$	$2.28.10^{-4}$	$3.981212.10^{5}$	$1.665.10^{-3}$	$12.408974.10^{6}$	$0.75.10^{-2}$
**4**	$1.902709.10^{4}$	$12.35.10^{-4}$	$2.274979.10^{5}$	$1.388.10^{-3}$	$3.8778043.10^{6}$	$1..10^{-2}$
**5**	$1.427032.10^{4}$	$6.83.10^{-4}$	$1.564048.10^{5}$	$1.11.10^{-3}$	$2.5205728.10^{6}$	$0.08125.10^{-2}$
**6**	$2.378387.10^{4}$	$1.76.10^{-4}$	$1.706234.10^{5}$	$1.203.10^{-3}$	$3.6839141.10^{6}$	$0.0875.10^{-2}$
**7**	$2.021629.10^{4}$	$4.23.10^{-4}$	$1.990606.10^{5}$	$1.018.10^{-3}$	$0.38778043.10^{6}$	$2.6875.10^{-2}$
**8**	$2.021629.10^{4}$	$5.2.10^{-4}$	$1.990606.10^{5}$	$0.925.10^{-3}$	$2.3266826.10^{6}$	$2.125.10^{-2}$
**9**	$2.140548.10^{4}$	$6.5.10^{-4}$	$8.531169.10^{5}$	$0.74.10^{-3}$	$3.4900239.10^{6}$	$1..10^{-2}$
**10**	$1.189193.10^{4}$	$7.8.10^{-4}$	$2.274979.10^{5}$	$0.833.10^{-3}$	$4.2655847.10^{6}$	$0.8125.10^{-2}$
**11**	$1.545951.10^{4}$	$10.08.10^{-4}$	$4.265585.10^{5}$	$1.48.10^{-3}$	$3.1022434.10^{6}$	$0.875.10^{-2}$
**12**	$1.902709.10^{4}$	$6.5.10^{-4}$	$1.706234.10^{5}$	$0.74.10^{-3}$	$3.8778043.10^{6}$	$1.5.10^{-2}$
**13**	$3.448661.10^{4}$	$2.28.10^{-4}$	$1.137489.10^{5}$	$1.295.10^{-3}$	$11.245633.10^{6}$	$1.5.10^{-2}$
**14**	$3.091903.10^{4}$	$4.23.10^{-4}$	$1.421862.10^{5}$	$0.814.10^{-3}$	$4.6533652.10^{6}$	$1.875.10^{-2}$
**15**	$3.56758.10^{4}$	$1.95.10^{-4}$	$2.417165.10^{5}$	$2.035.10^{-3}$	$4.6533652.10^{6}$	$1.875.10^{-2}$
**16**	$3.329741.10^{4}$	$4.88.10^{-4}$	$6.256191.10^{5}$	$2.59.10^{-3}$	$10.470072.10^{6}$	$0.875.10^{-2}$
**17**	$2.616225.10^{4}$	$6.83.10^{-4}$	$7.109308.10^{5}$	$2.775.10^{-3}$	$4.6533652.10^{6}$	$1.25.10^{-2}$
**18**	$2.735145.10^{4}$	$8.13.10^{-4}$	$3.412468.10^{5}$	$3.885.10^{-3}$	$2.3266826.10^{6}$	$0.75.10^{-2}$
**19**	$1.664871.10^{4}$	$7.48.10^{-4}$	$2.843723.10^{5}$	$1.48.10^{-3}$	$10.082291.10^{6}$	$0.75.10^{-2}$
**20**	$3.329741.10^{4}$	$14.3.10^{-4}$	$12.19957.10^{5}$	$6.475.10^{-3}$	$13.572315.10^{6}$	$1.5.10^{-2}$
**21**			$6.256191.10^{5}$	$2.22.10^{-3}$	$3.1022434.10^{6}$	$0.625.10^{-2}$
**22**			$6.540563.10^{5}$	$1.85.10^{-3}$	$9.6945108.10^{6}$	$1.375.10^{-2}$
**23**					$9.6945108.10^{6}$	$1.375.10^{-2}$
**24**					$9.6945108.10^{6}$	$1.5625.10^{-2}$
**25**					$7.7556086.10^{6}$	$0.75.10^{-2}$
**26**					$2.3266826.10^{6}$	$2.5.10^{-2}$
**27**					$1.1633413.10^{6}$	$1.0625.10^{-2}$
**28**					$1.1633413.10^{6}$	$8.125.10^{-2}$
**29**					$3.88.10^{6}$	$0.813.10^{-2}$
**30**					$1.1633413.10^{6}$	$1.125.10^{-2}$
**31**					$3.8778043.10^{6}$	$0.75.10^{-2}$
**32**					$2.714463.10^{6}$	$0.9375.10^{-2}$
**33**					$6.9800478.10^{6}$	$1.0625.10^{-2}$
**34**					$6.5922673.10^{6}$	$0.625.10^{-2}$
**35**					$5.428926.10^{6}$	$0.875.10^{-2}$
**36**					$3.4900239.10^{6}$	$0.6875.10^{-2}$
**37**					$2.3266826.10^{6}$	$0.6875.10^{-2}$
**38**					$4.2655847.10^{6}$	1.10^{-2}
**39**					$0.77556086.10^{6}$	$0.9375.10^{-2}$
**40**					$1.5511217.10^{6}$	1.10^{-2}
**41**					$12.021193.10^{6}$	$1.75.10^{-2}$
**42**					$4.2655847.10^{6}$	$0.9375.10^{-2}$
**43**					$3.4900239.10^{6}$	$0.5625.10^{-2}$
**44**					$9.6945108.10^{6}$	$2.8.10^{-2}$
**45**					$13.572315.10^{6}$	$1.5.10^{-2}$
**46**					$12.796754.10^{6}$	$1.75.10^{-2}$
**47**					$10.082291.10^{6}$	$1.875.10^{-2}$
**Variance**	**Var[N_{tr}]**	**Var[α_H]**	**Var[N_{tr}]**	**Var[α_H]**	**Var[N_{tr}]**	**Var[α_H]**
	$6.1271838.10^{7}$	$1.17.10^{-7}$	$7.91.10^{10}$	$1.71.10^{-6}$	$1.519.10^{13}$	$3.1.10^{-5}$
**Mean**	**E[N_{tr}]**	**E[α_H]**	**E[N_{tr}]**	**E[α_H]**	**E[N_{tr}]**	**E[α_H]**
	$2.267256.10^{4}$	$5.62.10^{-4}$	$3.155481.10^{5}$	$1.5.10^{-3}$	$4.4603989.10^{6}$	$1.1145.10^{-2}$
	**N_{tcoeff}=Var[N_{tr}]/ E[N_{tr}]**	**Nα_H=E[α_H]/ (Var[α_H]WL)**	**N_{tcoeff}=Var[N_{tr}]/ E[N_{tr}]**	**Nα_H=E[α_H]/ (Var[α_H]WL)**	**N_{tcoeff}=Var[N_{tr}]/ E[N_{tr}]**	**Nα_H=E[α_H]/ (Var[α_H]WL)**
	**$2.702466.10^{3}$**	**$1.19.10^{13}$**	**$2.505385.10^{5}$**	**$3.5.10^{11}$**	**$3.4046362.10^{6}$**	**$3.6.10^{10}$**
